# Supplementary material for: Systematic assessment of the achieved emission reductions of carbon crediting projects
Source: Nat Commun. 2024 Nov 14;15:9562. doi: 10.1038/s41467-024-53645-z (PMC11564741; doi:10.1038/s41467-024-53645-z)
Supplement: Supplementary file 1 — Supplementary Information [file 41467_2024_53645_MOESM1_ESM.pdf]

Supplementary Information for Systematic assessment of the achieved emission reductions of carbon crediting projects

Benedict S. Probst [*1,2,3], Malte Toetzke[1,4], Andreas Kontoleon[3], Laura Díaz Anadón[3,5], Jan C. Minx[6,7], Barbara K. Haya[8], Lambert Schneider[9], Philipp A. Trotter[10,11], Thales A.P. West[3,12], Annelise Gill-Wiehl[13], Volker H. Hoffmann[2]

[1] Net Zero Lab, Max Planck Institute for Innovation and Competition, Munich, Germany

[2] Group for Sustainability and Technology, ETH Zurich, Zurich, Switzerland

[3] Centre for Energy, Environment and Natural Resource Governance, Department of Land Economy, University of Cambridge, Cambridge, United Kingdom

[4] Public Policy for the Green Transition, Technical University Munich, Germany

[5] Harvard Kennedy School, Harvard University, Boston, United States

[6] Mercator Research Institute on Global Commons and Climate Change, Berlin, Germany

[7] Priestley International Centre for Climate, School of Earth and Environment, Leeds, United Kingdom

[8] Goldman School of Public Policy, University of California, Berkeley, USA

[9] Öko-Institut, Berlin, Germany

[10] Schumpeter School of Business and Economics, University of Wuppertal, Wuppertal, Germany

[11] Smith School of Enterprise and the Environment, University of Oxford, Oxford, United Kingdom

[12] Institute for Environmental Studies (IVM), Vrije Universiteit Amsterdam, Amsterdam, Netherlands

[13] Energy & Resources Group, University of California, Berkeley, United States

[*] Corresponding author, benedict.probst@ip.mpg.de

Table of Contents

Supplementary Table 1: Inclusion and exclusion criteria for studies. 3

Supplementary Table 2: Keywords used for search in SCOPUS. All articles downloaded: 26. Aug. 2022. 3

Supplementary Table 3: Keywords used for search in Web of Science. All articles downloaded: 26. Aug. 2022 5

Supplementary Table 4: Studies evaluating carbon crediting projects 6

Supplementary Table 5: Studies evaluating field interventions 8

Supplementary Table 6: OAR detailed approach for calculations 14

Supplementary Table 7: Studies that could not be integrated into quantitative framework 16

Supplementary Figure 1: ROSES flow diagram for systematic reviews. 17

Supplementary Table 1: Inclusion and exclusion criteria for studies.

	Population	Intervention	Comparator	Outcome	Study type
**Inclusion**	-	Voluntary, project-based activities that seek to reduce or remove emissions	Projects, land, or households that were not subject to the intervention (this can include historical data of the same project before it became a carbon mitigation project)	CO2e-emissions reduction (or comparable metric, such as deforestation)	Quantitative estimates based on randomised controlled trial or rigorous observational studies (which includes both modelling and empirical studies). These include working paper aimed at peer-reviewed journals and PhD theses
**Exclusion**	-	Non-voluntary activities (e.g., mandatory regulation) or non-project-based activities (e.g., carbon tax)	Without comparator	Without quantified impact of intervention	Qualitative studies

Supplementary Table 2: Keywords used for search in SCOPUS. All articles downloaded: 26. Aug. 2022.

	**Search Keywords in SCOPUS**
**1. Population**	-
**2. Intervention**	
Generic	"project-based mechanism*" OR "tradable emission* reduc* credit*" OR "carbon market*" OR "voluntary project*" OR "carbon W/5 offset*" OR "condition* payment*" OR "condition* cash transfer*" OR "economic* incentiv*" OR "clean development mechanism" OR "joint implementation mechanism" OR "kyoto protocol*"
Forestry and Land Use	
REDD+	"reduc* emission* from deforestation and forest degradation" OR "reduc* emission* from deforestat* and degradat*" OR "deforestat* reduc*" OR "payment* for ecosystem service*" OR "payment* for environmental services" OR "cash payment" OR "condition* pay*" OR "REDD+" OR "REDD"
Improved Forest Management	"forest*" W/5 ("manag*")
Afforestation / Reforestation	(payment* OR subsid*) W/5 (forest* OR plantat*) OR "afforest*" OR "reforest*"
Renewable Energy	
Wind	(wind) W/5 (farm* OR project* OR power OR energy)
Solar	(solar) W/5 (farm* OR project* OR power OR energy)

Hydro	(hydro*) W/5 (project OR power OR energy)
Biomass	(biomass) W/5 (project OR power OR energy)
Waste management	
Landfill / wastewater methane	“landfill” W/5 (“gas” OR “methane”) OR “wastewater” W/5 (“gas” OR “methane”)
Chemical processes	
Ozone depleting substances	“HFC-23” OR “SF6” OR “ozone” W/5 “deplet*” OR “regfrig*”
N2O destruction in nitric acid production	“N2O” AND “nitric*”
Household and community	
Cookstoves	*stove*
Industrial manufacturing	
Mine methane capture	“mine" AND "methane" AND "captur*
Natural gas electricity production	“natural” AND “gas” W/5 (project OR power OR energy)
Carbon capture and storage	
Carbon capture and enhanced oil recovery	“carbon” W/5 “captur*”
**3. Comparator**	
Generic	"control group*" OR "randomized trial" OR "evaluat*" OR "before-after-control-intervention" OR assess* OR impact* OR causal* OR "synthetic* control*" OR mechanism OR "quasi-experiment*" OR "Random* Control* Trial" OR "Random* trial*" OR "ex post" OR "ex post" OR baseline OR "difference*-in-difference*" OR "identification strategy" OR compliance OR "synthetic* match*" OR “confound* factors”
**4. Outcome**	
	“environment* integrity” OR (CO2 OR carbon OR SF6 OR HFC-23 OR “waste gas*” OR deforest* OR “forest*” OR “tree cover” OR “land cover” OR conservation OR “fuel” OR “greenhouse gas*” OR “wood*” OR “*coal”) W/5 (abat* OR “produc*” OR generat* OR lower* OR “conserv*” OR “impact*” OR “increas*” OR loss OR protect* OR “additional” OR “change” OR “decline*” OR “consum*” OR curb OR sav*)

Supplementary Table 3: Keywords used for search in Web of Science. All articles downloaded: 26. Aug. 2022

	**Search Keywords in WOS**
**1. Population**	-
**2. Intervention**	
Generic	“project-based mechanism*” OR “tradable emission* reduc* credit*” OR “carbon market*” OR “voluntary project*” OR “carbon NEAR/5 offset*“ OR “condition* payment*” OR “condition* cash transfer*” OR “economic* incentiv*” OR “clean development mechanism” OR “joint implementation mechanism” OR “kyoto protocol*”
Forestry and Land Use	
REDD+	"reduc* emission* from deforestation and forest degradation" OR "reduc* emission* from deforestat* and degradat*" OR "deforestat* reduc*" OR "payment* for ecosystem service*" OR "payment* for environmental services" OR "cash payment" OR “condition* pay*” OR "REDD+" OR "REDD"
Improved Forest Management	"forest*" NEAR/5 ("manag*") W/5 “improv*”
Afforestation / Reforestation	(payment* OR subsid*) NEAR/5 (forest* OR plantat*) OR “afforest*” OR “reforest*”
Renewable Energy	
Wind	(wind) NEAR/5 (farm* OR project* OR power OR energy)
Solar	(solar) NEAR/5 (farm* OR project* OR power OR energy)
Hydro	(hydro*) NEAR/5 (project OR power OR energy)
Biomass	(biomass) NEAR/5 (project OR power OR energy)
Waste management	
Landfill / wastewater methane	“landfill” NEAR/5 (“gas” OR “methane”) OR “wastewater” NEAR/10 (“gas” OR “methane”)
Chemical processes	
Ozone depleting substances	“HFC-23” OR “SF6” OR “ozone” NEAR/5 “deplet*” OR “regfrig*”
N2O destruction in nitric acid production	“N2O” AND “nitric*”
Household and community	
Cookstoves	*stove*
Industrial manufacturing	
Mine methane capture	“mine" AND "methane" AND "captur*”

Natural gas electricity production	“gas” NEAR/5 (project OR power OR energy)
Carbon capture and storage	
Carbon capture and enhanced oil recovery	“carbon” NEAR/5 “captur*”
**3. Comparator**	
Generic	"control group*" OR "randomized trial" OR "evaluat*" OR "before-after-control-intervention" OR assess* OR impact* OR causal* OR "synthetic* control*" OR mechanism OR "quasi-experiment*" OR "Random* Control* Trial" OR "Random* trial*" OR "ex post" OR "ex post" OR baseline OR "difference*-in-difference*" OR "identification strategy" OR compliance OR "synthetic* match*" OR “confound* factors”
**4. Outcome**	
	“environment* integrity” OR (CO2 OR carbon OR SF6 OR HFC-23 OR “waste gas*” OR deforest* OR “forest*” OR “tree cover” OR “land cover” OR conservation OR “fuel” OR “greenhouse gas*” OR “wood*” OR “*coal”) W/5 (abat* OR “produc*” OR generat* OR lower* OR “conserv*” OR “impact*” OR “increas*” OR loss OR protect* OR “additional” OR “change” OR “decline*” OR “consum*” OR curb OR sav*)

Supplementary Table 4: Studies evaluating carbon crediting projects

#	Authors	Title	DOI	Year	Region	Country	Sector	Project type
1	Chan and Huenteler	Financing Wind Energy Deployment in China through the Clean Development Mechanism	NA	2015	Asia	China	Renewable Energy	Wind
2	Calel et al.	Do Carbon Offsets Offset Carbon?	NA	2021	Asia	India	Renewable Energy	Wind
3	Schneider	Perverse incentives under the cdm: an evaluation of hfc-23 destruction projects	10.3763/cpol.2010.0096	2011	Multiple	Multiple	Chemical	HFC-23
4	Schneider and Kollmuss	Perverse effects of carbon markets on hfc-23 and sf6 abatement projects in russia	10.1038/nclimate2772	2015	Europe	Russia	Chemical	HFC-23, SF_6
5	Aung et al.	Health and climate-relevant pollutant concentrations from a carbon-finance approved cookstove	10.1021/acs.est.5b06208	2016	Asia	India	Household	Cook-stoves

		intervention in rural india						
6	Gill-Wiehl et al.	Cooking the books: Pervasive over-crediting from cookstoves offset methodologies	10.21203/rs.3.rs-2606020/v1	2022	Multipl e	Multipl e	Househol d	Cook-stoves
7	West et al.	Overstated carbon emission reductions from voluntary redd+ projects in the brazilian amazon	10.1073/pnas.2004334117	2020	Multipl e	Multipl e	Forestry	Avoided deforestati on
8	West et al.	Action needed to make carbon offsets from forest conservation work for climate change mitigation	10.1126/science.ade3535	2023	Multipl e	Multipl e	Forestry	Avoided deforestati on
9	Guizar-Coutiño et al. 2023	A global evaluation of the effectiveness of voluntary REDD+ projects at reducing deforestation and degradation in the moist tropics	10.1111/cobi.13970	2023	Multipl e	Multipl e	Forestry	Avoided deforestati on
10	Bomfim et al. 2023	Forest Carbon Accounting (in Quality assessment of REDD+ carbon credit projects. Berkeley Carbon Trading Project)	NA	2023	Multipl e	Multipl e	Forestry	Avoided deforestati on
11	Holm et al. 2023	Durability (in Quality assessment of REDD+ carbon credit projects. Berkeley Carbon Trading Project)	NA	2023	Multipl e	Multipl e	Forestry	Avoided deforestati on
12	Coffield et al. 2022	Using remote sensing to quantify the additional climate benefits of California forest carbon offset projects	10.1111/gcb.16380	2022	Norther n Americ a	USA	Forestry	IFM
13	Stapp et al. 2023	Little evidence of	10.1038/s43247-023-00984-2	2023	Norther n	USA	Forestry	IFM

		management change in California's forest offset program			America			
14	Badgley et al. 2021	Systematic over-crediting in California's forest carbon offsets program	https://doi.org/10.1111/gcb.15943	2021	Northern America	USA	Forestry	IFM

Supplementary Table 5: Studies evaluating field interventions

#	Authors	Title	DOI	Year	Region	Country	Sector	Project type
1	Gillenwater et al	Additionality of wind energy investments in the U.S. voluntary green power market	10.1016/j.renene.2013.10.003	2013	North America	United States of America	Renewable Energy	Wind
2	Ludwinski D., Moriarty K., Wydick B.	Environmental and health impacts from the introduction of improved wood stoves: evidence from a field experiment in guatemala	10.1007/s10668-011-9282-z	2011	Latin America	Guatemala	Household	Cookstoves
3	Jeuland M.A., Pattanayak S.K., Samaddar S., Shah R., Vora M.	Adoption and impacts of improved biomass cookstoves in rural rajasthan	10.1016/j.esd.2020.06.006	2020	Asia	India	Household	Cookstoves
4	Brooks N., Bhojvaid V., Jeuland M.A., Lewis J.J., Patange O., Pattanayak S.K.	How much do alternative cookstoves reduce biomass fuel use? evidence from north india	10.1016/j.reseneeco.2015.12.001	2016	Asia	India	Household	Cookstoves
5	Adrianzen, A.	Improved cooking stoves and firewood consumption: quasi-experimental evidence from the northern peruvian andes	10.1016/j.ecolecon.2013.02.010	2013	Latin America	Peru	Household	Cookstoves
6	Mekonen, A., Beyene, A., Bluffstone, R., Gebreegziabher, Z., Martinsson, P., Toman, M., Vieder, F.	Do improved biomass cookstoves reduce fuelwood consumption and carbon emissions? Evidence from a field experiment in rural Ethiopia	10.1016/j.ecolecon.2022.107467	2022	Africa	Ethiopia	Household	Cookstoves
7	Hanna R., Duflo E., Greenstone M.	Up in smoke: the influence of household behavior on the long-run impact	10.1257/pol.20140008	2016	Asia	India	Household	Cookstoves

		of improved cooking stoves						
8	Beltramo T., Levine D.I.	The effect of solar ovens on fuel use, emissions and health: results from a randomised controlled trial	10.1080/19439342.2013.775175	2013	Africa	Senegal	Household	Cookstoves
9	Bensch G., Peters J.	The intensive margin of technology adoption – Experimental evidence on improved cooking stoves in rural Senegal	10.1016/j.jhealeco.2015.03.006	2015	Africa	Senegal	Household	Cookstoves
10	Bensch and Peters	Alleviating Deforestation Pressures? Impacts of Improved Stove Dissemination on Charcoal Consumption in Urban Senegal	10.3368/le.89.4.676	2013	Africa	Senegal	Household	Cookstoves
11	Berkouwer, S., Dean, J.	Credit and attention in the adoption of profitable energy efficient technologies in Kenya	10.1257/aer.20210766	2022	Africa	Kenya	Household	Cookstoves
12	Carrilho C.D., Demarchi G., Duchelle A.E., Wunder S., Morsello C.	Permanence of avoided deforestation in a transamazon redd+ project (pará, brazil)	10.1016/j.ecolecon.2022.107568	2022	Latin America	Brazil	Forestry	Avoided deforestation
13	Simonet G., Subervie J., Ezzine-De-Blas D., Cromberg M., Duchelle A.E.	Effectiveness of a redd project in reducing deforestation in the brazilian amazon	10.1093/ajae/aay028	2018	Latin America	Brazil	Forestry	Avoided deforestation
14	Von Thaden J., Manson R.H., Congalton R.G., L√≥pez-Barrera F., Salcone J.	A regional evaluation of the effectiveness of mexico's payments for hydrological services	10.1007/s10113-019-01518-3	2019	Latin America	Mexico	Forestry	Avoided deforestation
15	Jayachandran S., De Laat J., Lambin E.F., Stanton C.Y., Audy R., Thomas N.E.	Cash for carbon: a randomized trial of payments for ecosystem services to reduce deforestation	10.1126/science.aan0568	2017	Africa	Uganda	Forestry	Avoided deforestation
16	Montoya-Zumaeta J., Rojas E., Wunder S.	Adding rewards to regulation: the impacts of watershed conservation on land cover and household	10.1371/journal.pone.0225367	2019	Latin America	Peru	Forestry	Avoided deforestation

		wellbeing in moyobamba, peru						
17	Le Velly, G; Sauquet, A; Cortina-Villar, S	PES impact and leakages over several cohorts: the case of the psa-h in yucatan, mexico	10.3368/le.93.2.230	2017	Latin America	Mexico	Forestry	Avoided deforestation
18	Mohebalian P.M., Aguilar F.X.	Design of tropical forest conservation contracts considering risk of deforestation	10.1016/j.landusepol.2017.11.008	2018	Latin America	Ecuador	Forestry	Avoided deforestation
19	Jones K.W., Holland M.B., Naughton-Treves L., Morales M., Suarez L., Keenan K.	Forest conservation incentives and deforestation in the ecuadorian amazon	10.1017/s0376892916000308	2017	Latin America	Ecuador	Forestry	Avoided deforestation
20	Costedoat S., Corbera E., Ezzine-de-Blas D., Honey-Ros√©s J., Baylis K., Castillo-Santiago M.A.	How effective are biodiversity Conservation payments in mexico?	10.1371/journal.pone.0119881	2015	Latin America	Mexico	Forestry	Avoided deforestation
21	Clements T., Milner-Gulland E.J.	Impact of payments for environmental services and protected areas on local livelihoods and forest conservation in northern cambodia	10.1111/cobi.12423	2014	Asia	Cambodia	Forestry	Avoided deforestation
22	Ramirez-Reyes C., Sims K.R.E., Potapov P., Radeloff V.C.	Payments for ecosystem services in mexico reduce forest fragmentation	10.1002/eap.1753	2018	Latin America	Mexico	Forestry	Avoided deforestation
23	Honey-Roses J., Baylis K., Ramirez M.I.	A spatially explicit estimate of avoided forest loss	10.1111/j.1523-1739.2011.01729.x	2018	Latin America	Mexico	Forestry	Avoided deforestation
24	Arriagada R.A., Ferraro P.J., Sills E.O., Pattanayak S.K., Cordero-Sancho S.	Do payments for environmental services affect forest cover? a farm-level evaluation from costa rica	10.3368/le.88.2.382	2012	Latin America	Costa Rica	Forestry	Avoided deforestation
25	Ruggiero P.G.C., Metzger J.P., Reverberi Tambosi L., Nichols E.	Payment for ecosystem services programs in the brazilian atlantic forest: effective but not enough	10.1016/j.landusepol.2018.11.054	2019	Latin America	Brazil	Forestry	Avoided deforestation
26	Robalino, J; Pfaff, A; Sandoval,	Can we increase the impacts from payments	10.1016/j.forpol.2021.102577	2021	Latin America	Costa Rica	Forestry	Avoided deforestation

	C; Sanchez-Azofeifa, GA	for ecosystem services? impact rose over time in costa rica, yet spatial variation indicates more potential						
27	Robalino et al	Evaluating Interactions of Forest Conservation Policies on Avoided Deforestation	doi.org/10.1371/journal.pone.0124910	2015	Latin America	Costa Rica	Forestry	Avoided deforestation
28	Bos A.B., Duchelle A.E., Angelsen A., Avitabile V., De Sy V., Herold M., Joseph S., De Sassi C., Sills E.O., Sunderlin W.D., Wunder S.	Comparing methods for assessing the effectiveness of subnational redd+ initiatives	10.1088/1748-9326/aa7032	2017	Multiple	Multiple	Forestry	Avoided deforestation
29	Correa J., Cisneros E., B√∂rner J., Pfaff A., Costa M., Raj√£o R.	Evaluating redd+ at subnational level: amazon fund impacts in alta floresta, brazil	10.1016/j.forpol.2020.102178	2020	Latin America	Brazil	Forestry	Avoided deforestation
30	Ellis E.A., Sierra-Huelsz J.A., Ceballos G.C.O., Binnq√ºist C.L., Cerd√°n C.R.	Mixed effectiveness of redd+ subnational initiatives after 10 years of interventions on the yucatan peninsula, mexico	10.3390/f11091005	2020	Latin America	Mexico	Forestry	Avoided deforestation
31	Roopsind A., Sohngen B., Brandt J.	Evidence that a national redd program reduces tree cover loss and carbon emissions in a high forest cover, low deforestation country	10.1073/pnas.1904027116	2019	Latin America	Guyana	Forestry	Avoided deforestation
32	Sims K.R.E., Alix-Garcia J.M.	Parks versus pes: evaluating direct and incentive-based land conservation in mexico	10.1016/j.jeem.2016.11.010	2016	Latin America	Mexico	Forestry	Avoided deforestation
33	Alix-Garcia J.M., Sims K.R.E., Yañez-Pagans P.	Only one tree from each seed? environmental effectiveness and poverty alleviation in mexico's payments for ecosystem services program	10.1257/pol.20130139	2015	Latin America	Mexico	Forestry	Avoided deforestation

34	Alix-Garcia J.M., Shapiro E.N., Sims K.R.E.	Forest conservation and slippage: evidence from mexico's national payments for ecosystem services program	10.3368/le.88.4.613	2012	Latin America	Mexico	Forestry	Avoided deforestation
35	Chervier C., Costedoat S.	Heterogeneous impact of a collective payment for environmental services scheme on reducing deforestation in cambodia	10.1016/j.worlddev.2017.04.014	2017	Asia	Cambodia	Forestry	Avoided deforestation
36	Jones K.W., Mayer A., Von Thaden J., Berry Z.C., López-Ramírez S., Salcone J., Manson R.H., Asbjornsen H.	Measuring the net benefits of payments for hydrological services programs in mexico	10.1016/j.ecolecon.2020.106666	2020	Latin America	Mexico	Forestry	Avoided deforestation
37	Giudice R., Börner J., Wunder S., Cisneros E.	Selection biases and spillovers from collective conservation incentives in the peruvian amazon	10.1088/1748-9326/aafc83	2019	Latin America	Peru	Forestry	Avoided deforestation
38	Jones K.W., Lewis D.J.	Estimating the counterfactual impact of conservation programs on land cover outcomes: the role of matching and panel regression techniques	10.1371/journal.pone.0141380	2015	Latin America	Ecuador	Forestry	Avoided deforestation
39	Cisneros E., Börner J., Pagiola S., Wunder S.	Impacts of conservation incentives in protected areas: the case of bolsa floresta, brazil	10.1016/j.jeem.2021.102572	2022	Latin America	Brazil	Forestry	Avoided deforestation
40	Etchart N., Freire J.L., Holland M.B., Jones K.W., Naughton-Treves L.	What happens when the money runs out? forest outcomes and equity concerns following ecuador's suspension of conservation payments	10.1016/j.worlddev.2020.105124	2020	Latin America	Brazil	Forestry	Avoided deforestation
41	Pagiola S., Honey-Rosés J., Freire-González J.	Evaluation of the permanence of land use change induced by payments for environmental services in quindío, colombia	10.1371/journal.pone.0147829	2016	Latin America	Colombia	Forestry	Avoided deforestation

42	Fiorini A.C., Mullally C., Swisher M., Putz F.E.	Forest cover effects of payments for ecosystem services: evidence from an impact evaluation in brazil	10.1016/j.ecolecon.2019.106522	2020	Latin America	Brazil	Forestry	Avoided deforestation
43	Montoya-Zumaeta J.G., Wunder S., Rojas E., Duchelle A.E.	Does redd+ complement law enforcement? evaluating impacts of an incipient initiative in madre de dios, peru	10.3389/ffgc.2022.870450	2022	Latin America	Peru	Forestry	Avoided deforestation
44	Sharma B.P., Karky B.S., Nepal M., Pattanayak S.K., Sills E.O., Shyamsundar P.	Making incremental progress: impacts of a redd+ pilot initiative in nepal	10.1088/1748-9326/aba924	2020	Asia	Nepal	Forestry	Avoided deforestation
45	Zhou T., Shen W., Qiu X., Chang H., Yang H., Yang W.	Impact evaluation of a payments for ecosystem services program on vegetation quantity and quality restoration in inner mongolia	10.1016/j.jenvman.2021.114113	2022	Asia	Inner Mongolia	Forestry	Avoided deforestation
46	España F., Arriagada R., Melo O., Foster W.	Forest plantation subsidies: impact evaluation of the chilean case	10.1016/j.forpol.2022.102696	2022	Latin America	Chile	Forestry	Afforestation/Reforestation
47	Fu G., Uchida E., Shah M., Deng X.	Impact of the grain for green program on forest cover in china	10.1080/21606544.2018.1552626	2019	Asia	China	Forestry	Avoided deforestation
48	Hayes T., Murtinho F., Wolff H., López-Sandoval M.F., Salazar J.	Effectiveness of payment for ecosystem services After loss and uncertainty of compensation	10.1038/s41893-021-00804-5	2022	Latin America	Ecuador	Forestry	Avoided deforestation
49	Linkie M., Smith, R., Zhu Y., Martyr, D., et al	Evaluating Biodiversity Conservation around a Large Sumatran Protected Area	10.1111/j.1523-1739.2008.00906.x	2007	Asia	Indonesia	Forestry	Avoided deforestation
50	Erbaugh J.T.	Impermanence and failure: the legacy of conservation-based payments in sumatra, indonesia	10.1088/1748-9326/ac6437	2022	Asia	Indonesia	Forestry	Avoided deforestation
51	Cuenca P., Robalino J., Arriagada R., Echeverría C.	Are government incentives effective for avoided deforestation in	10.1371/journal.pone.0203545	2018	Latin America	Ecuador	Forestry	Avoided deforestation

		the tropical andean forest?							

For IFM and wind power, we do not find statistically significant results. Yet, these projects could still lead to emissions reductions but may be statistically underpowered. We, therefore, inspect the point estimates of these studies and find that for two of them, the point estimates are either inconclusive or indicate an emissions increase (rather than a reduction). For these projects, we therefore assume an OAR of 0.

Supplementary Table 6: OAR detailed approach for calculations

Study	Sector, project type	Transformation approach
West et al (2020, 2023), Guizar-Coutinho (2023)	Forestry, Avoided deforestation	All studies report changes in deforestation (and, for Guizar-Coutiño degradation) rates between projects sites and those not covered. See equation (3) in main manuscript for approach to convert numbers.<br><br>West et al. analyse 36 projects, whereas Guizar-Coutinho et al. analyses 40 projects. To compute the OAR, we needed to post-process the existing study results. Estimates of the likely actual emission reductions were based on the average carbon stock per hectare of the most representative forest stratum and post-deforestation land-use class in the project area, obtained from the official project descriptions, multiplied by the estimated forest area prevented from deforestation (ha). The estimates of the area prevented from deforestation by Guizar-Coutiño et al. are based on the difference between control and project deforestation (%) multiplied by the project area (ha). Our estimate of the likely actual emission reductions were then compared to the volumes of credits issued to the projects. To make the estimates of the studies comparable, we consider the same time periods. As Guizar-Coutiño et al.[1] use shorter timeframes (5 years) than the West et al.[2,3] studies, we use the time periods covered in Guizar-Coutiño and re-calculate the original estimates from the West studies for the same time frames. To calculate the issued credits during the covered time frame, we

		proceed in two steps. First, we compile a list with the total volume of credits issued for each VCS project covered in the studies. Second, we compute the average yearly issued credit volume and calculate the total average 5-year issuance for each project.
Stapp et al., Coffield et al.	Forestry, Improved Forest Management	Both studies assess the changes in disturbance/harvesting rates on project and non-project areas. As both studies find no statistically significant change to harvesting rates, we assume an OAR of 0.
Aung et al.	Household, Cookstoves	Aung finds no statistically significant change in biomass consumption in treated and non-treated households. While Aung only covers one factor of over/under-crediting (in contrast to Gill-Wiehl, which covers all relevant factors), we assume an OAR of 0, because if there are no changes to biomass consumption, the study results would not change, even if the study considered other over/under-crediting factors.
Gill-Wiehl et al.	Household, Cookstoves	The study directly estimates the OAR, which is the inverse of the over-crediting factor provided in the study (1/over-crediting). Please note that to determine the total volume of issued credits from the projects in Figure 5, we update the information from the manuscript by using the Berkeley Voluntary Registry Offsets database v9 version, which covers credits up to Nov 2023[4].
Schneider 2011	Chemicals, HFC-23	For two HFC-23 CDM plants it was observed that they generated less waste gas during periods in which they could not issue carbon credits (Schneider 2011). For these two plants we determine the offset achievement ratio for two different scenarios: (1) assuming that the plants would have operated at the waste generation rate observed in the period where they were not eligible for crediting and (2) assuming that the plants would have operated at historical waste generation rates observed prior to crediting. The average value resulting from these scenarios is used as the central estimate for the OAR. The analysis is based on data used in the underlying paper

		and does not include data on monitoring reports that were published later on. The total number of credits issued to the plants is drawn from the UNFCCC CDM database.
Schneider and Kollmuss 2015	Chemicals, HFC-23/SF6	For the three projects abating HFC-23 and/or SF6 waste gas in Russia we determine the offset achievement ratio based on the three scenarios provided in the supplementary information of the underlying paper. The total issuance of 54 million emission reduction units (ERUs) is also taken from the paper.
Chan and Huenteler; Calel et al.	Renewable Energy, Wind	Chan and Huenteler do not find statistically significant differences in the viability between projects that sold credits under the CDM and those that did not. We, therefore, assume an OAR of 0. Calel et al. could not be integrated into our quantitative framework as they only provide upper bound estimates (see discussion in manuscript).

Supplementary Table 7: Studies that could not be integrated into quantitative framework

**Study**	**Reason for non-integration**
Calel et al.	Study only identifies the most obvious cases of non-additionality, but the authors do not provide a central estimate.
Bomfim et al. 2023	Study assesses problems relating to carbon accounting. We felt that that it would be inadequate to use the findings from the 12 sample projects and apply it to all covered projects in the review.
Holm et al. 2023	Study assesses the durability of issued carbon credits, identifying that reversal risks are likely underestimated. Yet, as our review focuses on ex-post studies, we were unable to integrate the forward-looking methodology into our quantitative framework.
Badgley et al. 2021	Study assesses over-crediting from IFM projects in California. Yet, the study does not directly analyze counterfactual project areas, and thus, cannot be integrated in our framework.

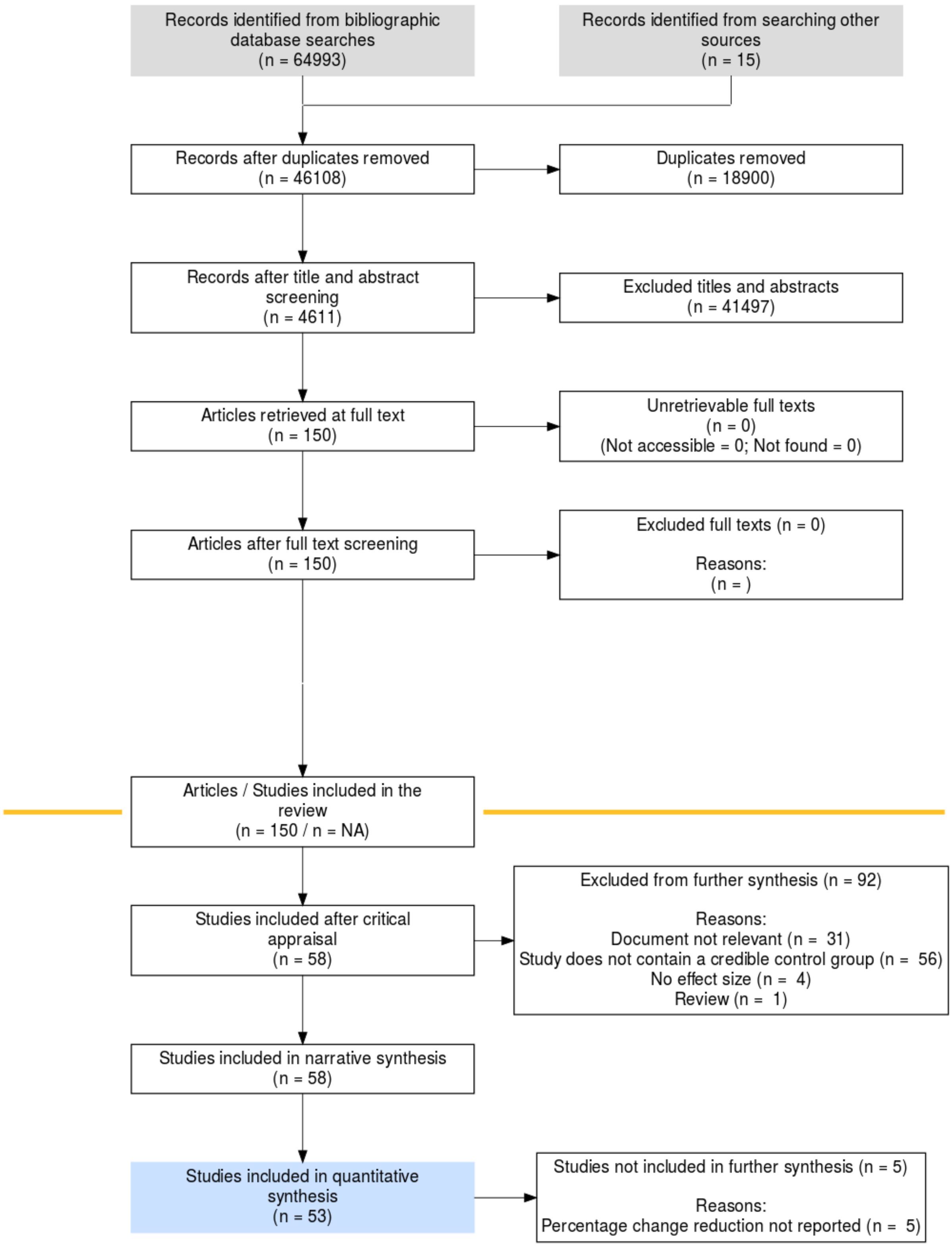

Supplementary Figure 1: ROSES flow diagram for systematic reviews.

The figure shows the screening process used in the systematic assessment, starting from a full set of potentially relevant studies (64,993) to the final set of studies that were included in the quantitative synthesis. Please note that 12 additional studies were included due to manual search not shown in figure.
