# Supplementary material for: Systematic assessment of the achieved emission reductions of carbon crediting projects
Source: Nat Commun. 2024 Nov 14;15:9562. doi: 10.1038/s41467-024-53645-z (PMC11564741; doi:10.1038/s41467-024-53645-z)
Supplement: Supplementary file 3 — Reporting summary [file 41467_2024_53645_MOESM3_ESM.pdf]

nature portfolio

Corresponding author(s): *Double-anonymous peer review submissions: write DAPR and your manuscript number here instead of author names.*

Last updated by author(s): *YYYY-MM-DD*

Reporting Summary

Nature Portfolio wishes to improve the reproducibility of the work that we publish. This form provides structure for consistency and transparency in reporting. For further information on Nature Portfolio policies, see our Editorial Policies and the Editorial Policy Checklist.

Statistics

For all statistical analyses, confirm that the following items are present in the figure legend, table legend, main text, or Methods section.

n/a	Confirmed	
☐	☒	The exact sample size (*n*) for each experimental group/condition, given as a discrete number and unit of measurement
☐	☒	A statement on whether measurements were taken from distinct samples or whether the same sample was measured repeatedly
☐	☒	The statistical test(s) used AND whether they are one- or two-sided<br>*Only common tests should be described solely by name; describe more complex techniques in the Methods section.*
☒	☐	A description of all covariates tested
☒	☐	A description of any assumptions or corrections, such as tests of normality and adjustment for multiple comparisons
☐	☒	A full description of the statistical parameters including central tendency (e.g. means) or other basic estimates (e.g. regression coefficient) AND variation (e.g. standard deviation) or associated estimates of uncertainty (e.g. confidence intervals)
☒	☐	For null hypothesis testing, the test statistic (e.g. *F*, *t*, *r*) with confidence intervals, effect sizes, degrees of freedom and *P* value noted<br>*Give P values as exact values whenever suitable.*
☒	☐	For Bayesian analysis, information on the choice of priors and Markov chain Monte Carlo settings
☒	☐	For hierarchical and complex designs, identification of the appropriate level for tests and full reporting of outcomes
☒	☐	Estimates of effect sizes (e.g. Cohen's *d*, Pearson's *r*), indicating how they were calculated

Our web collection on statistics for biologists contains articles on many of the points above.

Software and code

Policy information about availability of computer code

Data collection	We relied on the AI-supported systematic review tool AS Review to order our complete study set in order of probable relevance. Estimates were manually extracted from relevant studies.
Data analysis	Analysis was conducted in Excel (version 16.88).

For manuscripts utilizing custom algorithms or software that are central to the research but not yet described in published literature, software must be made available to editors and reviewers. We strongly encourage code deposition in a community repository (e.g. GitHub). See the Nature Portfolio guidelines for submitting code & software for further information.

Data

Policy information about availability of data

All manuscripts must include a data availability statement. This statement should provide the following information, where applicable:

- Accession codes, unique identifiers, or web links for publicly available datasets
- A description of any restrictions on data availability
- For clinical datasets or third party data, please ensure that the statement adheres to our policy

All data collected is available as a data book published with the original study

Research involving human participants, their data, or biological material

Policy information about studies with human participants or human data. See also policy information about sex, gender (identity/presentation), and sexual orientation and race, ethnicity and racism.

Reporting on sex and gender	NA
Reporting on race, ethnicity, or other socially relevant groupings	NA
Population characteristics	NA
Recruitment	NA
Ethics oversight	NA

Note that full information on the approval of the study protocol must also be provided in the manuscript.

Field-specific reporting

Please select the one below that is the best fit for your research. If you are not sure, read the appropriate sections before making your selection.

☐ Life sciences ☒ Behavioural & social sciences ☐ Ecological, evolutionary & environmental sciences

For a reference copy of the document with all sections, see nature.com/documents/nr-reporting-summary-flat.pdf

Behavioural & social sciences study design

All studies must disclose on these points even when the disclosure is negative.

Study description	Systematic review
Research sample	Data was compiled from 65 studies, of which 14 contained detailed information on carbon crediting projects
Sampling strategy	After having defined the keywords and inclusion and exclusion criteria, we ran the search on SCOPUS and Web of Science as well as manual searches on Google Scholar. As our keywords were inclusive and we did not impose restrictions based on the scientific discipline, publication date or study design, our search led to a large set of potentially relevant studies (64,993). After removing duplicates, our search returned 46,108 studies. We relied on the AI-supported systematic review tool AS Review9 to order our complete study set in order of probable relevance. AS Review is a software tool that allows for more efficient screening of titles and abstracts.
Data collection	A team of two researchers manually screened and labelled (relevant / not relevant) the title and abstract of the first 4,611 studies ordered by probable relevance based on AS Review. While we cannot rule out that our prioritised screening approach omitted some relevant studies, our screening approach is in line with other AI-supported systematic reviews26. To ascertain that we did not miss critical studies, the author team also searched manually for relevant studies. Out of the reviewed studies, 150 studies were flagged for full-text review. Of these 150 studies, 58 were excluded after critical appraisal due to non-relevance (31), absence of a credible comparator (56), no effect size (4) and review (1). The final set included 65 studies, of which 12 studies were added based on a manual search. Then, two researchers independently extract the reported effect sizes from individual projects and other relevant aspects of the study detailed in our Codebook.
Timing	All studies were downloaded on the 22nd of August 2022. During the review process, we reran the search to see whether any relevant research had been published in the meantime and did manual searches to complement the existing set of studies.
Data exclusions	To operationalise the research question, we developed the search strings iteratively. We started with relevant studies known to the author team to define keywords. We searched for academic studies that evaluate voluntary, project-based activities that seek to reduce emissions or enhance removals. We excluded studies that evaluate non-voluntary activities such as mandatory regulations or non-project-based activities (e.g., other forms of carbon pricing such as carbon taxes). We focus on studies that evaluate project impact against a credible comparator. This comparator can include projects, land, or households that were.
Non-participation	NA
Randomization	NA

Reporting for specific materials, systems and methods

We require information from authors about some types of materials, experimental systems and methods used in many studies. Here, indicate whether each material, system or method listed is relevant to your study. If you are not sure if a list item applies to your research, read the appropriate section before selecting a response.

Materials & experimental systems

n/a	Involved in the study
☒	☐ Antibodies
☒	☐ Eukaryotic cell lines
☒	☐ Palaeontology and archaeology
☒	☐ Animals and other organisms
☒	☐ Clinical data
☒	☐ Dual use research of concern
☒	☐ Plants

Methods

n/a	Involved in the study
☒	☐ ChIP-seq
☒	☐ Flow cytometry
☒	☐ MRI-based neuroimaging

Plants

Seed stocks	NA
Novel plant genotypes	NA
Authentication	NA
