# Supplementary material for: Systematic assessment of the achieved emission reductions of carbon crediting projects
Source: Nat Commun. 2024 Nov 14;15:9562. doi: 10.1038/s41467-024-53645-z (PMC11564741; doi:10.1038/s41467-024-53645-z)
Supplement: Supplementary file 5 — Supplementary data [file 41467_2024_53645_MOESM5_ESM.pdf]

## Description of Additional Supplementary Files

**File name:** Supplementary Data

**Description:** This Excel file includes detailed data supporting Probst et al.'s (2024) study on emission reductions from carbon crediting projects. It contains tabs with offset achievement ratios across all sectors examined, with project-level data provided when available.
